# An observational study using ultrasound to assess physiological changes following fluid bolus administration in paediatric sepsis in the emergency department

**DOI:** 10.1186/s12887-016-0634-6

**Published:** 2016-07-15

**Authors:** Elliot Long, Ed Oakley, Franz E. Babl, Trevor Duke, Paediatric Research in Emergency Departments International Collaborative (PREDICT)

**Affiliations:** Department of Emergency Medicine, The Royal Children’s Hospital, 50 Flemington Road, 3052 Parkville, VIC Australia; Murdoch Children’s Research Institute, 50 Flemington Road, Parkville, Australia; Department of Paediatrics, Faculty of Medicine, Dentistry, and Health Sciences, University of Melbourne, Victoria, Australia; Paediatric Intensive Care Unit, The Royal Children’s Hospital, 50 Flemington Road, Parkville, VIC Australia

**Keywords:** Sepsis, Fluid therapy, Ultrasound, Cardiac index, Extravascular lung water, Paediatric

## Abstract

**Background:**

Fluid bolus administration is widely recommended as part of the initial treatment of paediatric sepsis, though the physiological benefits and harms are unclear. The primary aim of this study is to determine the effect of fluid bolus administration on cardiac index (CI). Secondary aims are to determine the effect of fluid bolus administration on extra-vascular lung water (EVLW), whether fluid responsiveness can be predicted by inferior vena cava (IVC) collapsibility, and whether fluid responsiveness correlates with changes in vital signs.

**Methods/design:**

A prospective observational study of children presenting to the Emergency Department of The Royal Children’s Hospital with clinically diagnosed sepsis requiring fluid bolus administration. Prior to fluid bolus administration, an echocardiogram, lung ultrasound, and IVC ultrasound will be performed, and vital signs recorded. These will be repeated 5 min after and 60 min after fluid bolus administration. Recorded echocardiograms and lung/IVC ultrasound will be evaluated independently by a paediatric cardiologist and paediatric emergency physician, respectively, blinded to the patient identity and time of examination relative to time of fluid bolus administration. Fifty patients will be enrolled in the study based on a precision based sample size calculation. Results will be analysed for change in CI and change in EVLW 5 min after and 60 min after fluid bolus administration compared to baseline, IVC collapsibility as a predictor of fluid responsiveness, and the relationship between fluid responsiveness and changes in vital signs.

**Discussion:**

This study will explore assumptions about the effect of fluid boluses on CI in children with sepsis, and will provide evidence for secondary effects on other organ systems. This may lead to novel methods for assessment and decision making in the initial resuscitation of paediatric sepsis in clinical and research settings, and will likely influence the design of future interventional studies in this arena.

**Trial registration:**

The study is registered with the Australian and New Zealand Clinical Trials Registry (ACTRN12614000824662; 04 August 2014).

## Background

Sepsis is the final common pathway and mode of death for many paediatric infectious processes, globally accounting for over 6 million childhood deaths per year, mostly in underdeveloped countries [1]. Case fatality rates are 5–10 % in industrialised countries, and 20–30 % in low and middle income countries [2, 3]. Despite being common and serious, the therapies for the initial resuscitation of paediatric sepsis are based on little or no evidence. In particular, fluid resuscitation therapy (FRT) is widely recommended for initial sepsis resuscitation [4, 5], but there is limited understanding of its potential beneficial and harmful effects, how the balance of benefits and harms may be influenced by patient and disease factors, and how this balance may be incorporated into treatment guidelines for translation into clinical use.

Fluid resuscitation was initially described in patients with severe dehydration during cholera epidemics in the early 19^th^ century [6], and subsequently applied to trauma patients with haemorrhagic shock [7]. Both of these disease states are characterised by intravascular volume depletion, and replacement of this intravascular volume with the same type of fluid that was lost helps to restore homeostasis. Fluid resuscitation for sepsis was popularised in 2001, when early goal-directed therapy (EGDT) was shown to improve survival in adults with septic shock when instituted in the emergency department (ED) [8]. This approach involved aggressive fluid resuscitation to achieve threshold central venous pressure (CVP) values, followed by early initiation of vasoactive agents for ongoing hypotension, and transfusion of blood for low central venous oxygen saturations (ScvO2). As the initial therapy in EGDT, aggressive fluid resuscitation was the most commonly applied in practice, and the foundation upon which subsequent therapies were based. Since the publication of the EGDT protocol, the harms from aggressive fluid resuscitation and cumulative positive fluid balance have become apparent [9–16], and the benefit of EGDT compared to standard care has been called into question [17–19]. In particular, the Fluid Expansion As Supportive Therapy (FEAST) study found that FRT in Sub-Saharan African children with fever and signs of poor perfusion increased mortality compared to no FRT [20]. This raises serious questions about the role of FRT in the management of sepsis, and our understanding of the pathophysiological mechanisms underlying FRT in conditions where no volume loss has occurred.

Aggressive FRT with the aim of increasing stroke volume, with the assumption that this will improve end-organ perfusion and cellular oxygen use, may be physiologically flawed [21]. FRT into high-capacitance veins may in fact increase cardiac filling pressures faster than venous pressure due to limited diastolic compliance of the heart, thus reducing the pressure gradient driving venous return [22–24]. Increased venous pressure following FRT may reduce the gradient driving vital organ perfusion (MAP-CVP) [25]. Microcirculatory dysfunction through damage to the endothelial glycocalyx may be mediated partly by natriuretic peptides released as cardiac filling pressures increase following FRT [26–30]. The net effect of FRT in sepsis may be that it contributes significantly to end-organ oedema and dysfunction, and the progression from SIRS to multi-organ dysfunction and death [9, 10, 12, 13, 31, 32].

Limited published literature on the effect of FRT on cardiac output (CO) suggests that any effect is modest and transient. In a study of 20 adults with circulatory shock, the mean increase in CO immediately following FRT was 16 %, which decreased to 6 % above baseline after sixty minutes [33]. In a separate study, 32 adults with septic shock were found to have a mean increase in CO of 15 % immediately after FRT, which returned to 3 % above baseline 40 min after FRT (*p* < 0.001) [34]. There is no published literature on the effect of FRT on cardiac index (CI = CO/body surface area (BSA)) in children. The ability to predict the response to FRT allows the clinician to restrict this therapy only to those that are fluid responsive. Fluid responsiveness is considered an increase in cardiac output of 10-15 % in response to a fluid challenge (250–500 ml in adults) [35]. In the undifferentiated adult ICU population, as few as 50 % of patients may be fluid responsive [35]. The remainder receive all of the potential harms from FRT with none of the potential benefits. IVC ultrasound is a dynamic method for assessing fluid responsiveness using cardiopulmonary interactions [36]. In spontaneously ventilating adults, IVC collapsibility of >40 % has been shown to be predictive of fluid responsiveness, while IVC collapsibility of <15 % is predictive of being fluid unresponsive [37]. No study has validated IVC collapsibility as a predictor of fluid responsiveness in children.

We set out to determine the effect of FRT on CI 5 min after and 60 min after fluid bolus administration in paediatric sepsis. Secondary aims include: the effect of FRT on extra-vascular lung water (EVLW), whether fluid responsiveness can be predicted by inferior vena cava (IVC) collapsibility, and whether fluid responsiveness correlates with changes in vital signs.

## Methods/design

### Study design and setting

The study is designed as a prospective observational study of children with sepsis. It will be conducted at a single paediatric ED in The Royal Children’s Hospital (RCH), Melbourne, Australia. RCH is a tertiary children’s hospital with an annual ED census of >85,000 children less than 18 years of age.

### Inclusion and exclusion criteria

Inclusion and exclusion are listed in Table 1. Inclusion criteria are based on international consensus definitions for the diagnosis of sepsis in children [38].

**Table 1:** Inclusion and exclusion criteria

Inclusion criteria- all of: Suspected infection Temperature >38 °C or <36 °C Systemic Inflammatory Response Syndrome Tachycardia (temperature corrected heart rate >2 SD above normal for age^a^) *or* Tachypnoea (respiratory rate >2 SD above normal for age) Plan to administer a standardised fluid bolus by the treating clinician Standardised fluid bolus: 20 ml/kg of 0.9 % saline administered via manual push/pull or pressure bag (NOT by infusion pump). Exclusion criteria- any of: Underlying uncorrected structural cardiac disease Non-curative goals of therapy Non-English speaking	

^a^heart rate was corrected by 10 beats per minute for every degree celcius above 38^o^ [55]

### Study procedure

**Fig. 1:**
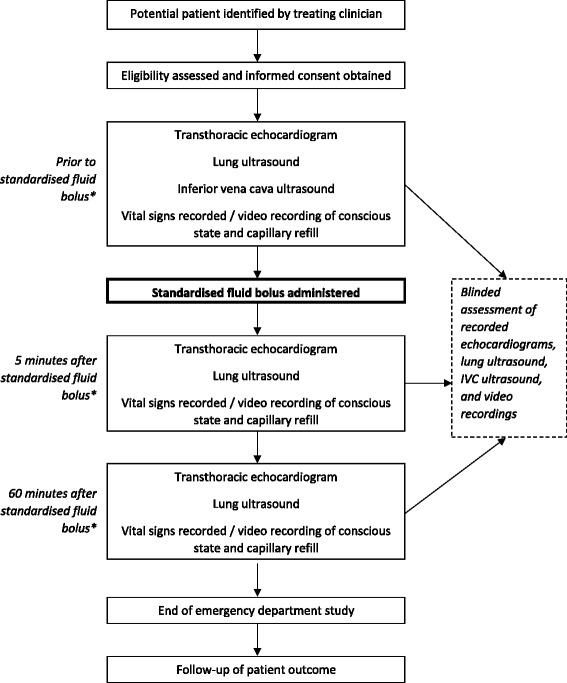
Schedule of observations recorded during the 60 min study period. *a standardised fluid bolus was defined as 20 ml/kg of 0.9 % saline

Patients who fulfil the eligibility criteria and whose parents provide written informed consent will be entered into the study. Prior to administration of a standardised fluid bolus, transthoracic echocardiography, lung and IVC ultrasound will be performed, and vital signs recorded (Fig. 1). Five minutes and 60 min after administration of a standardised fluid bolus, the same measurements will be taken. All ultrasound measurements will be taken by the Principal Investigator (PI), who has a Post Graduate Certificate in Echocardiography through The University of Melbourne, Australia. Recorded echocardiograms will subsequently be assessed by a blinded paediatric cardiologist. Lung ultrasound and IVC ultrasound will be assessed by a blinded paediatric emergency physician with a Diploma in Diagnostic Ultrasound (Australian Society for Ultrasound in Medicine). Vital signs that will be recorded from the patients monitor (Phillips MP70, Phillips Healthcare, Andover, MA) include: heart rate (HR), respiratory rate (RR), blood pressure (BP), and peripheral oxyhaemoglobin saturation (SpO2). Additionally, patient temperature in degrees celcius will be recorded and a 10 s video recording of the patient will be taken at each time point and used to assess conscious state, capillary refill, and respiratory effort. These video recordings will be scored in terms of Glasgow Coma Scale for conscious state, time in seconds for capillary refill, and Silverman Score for respiratory effort [39] by a blinded paediatric emergency physician. Final outcomes will be obtained from the patient medical record following discharge or death.

### Study flow chart

#### Outcome measures

*The primary outcome measure* is the percentage change in CI 5 and 60 min after standardised fluid bolus administration compared to baseline.

*Secondary outcome measures* include the change in EVLW after standardised fluid bolus administration, whether IVC collapsibility is predictive of fluid responsiveness, and whether the observed change in cardiac output following standardised fluid bolus administration is correlated with changes in vital signs and clinical observations. We also collected the following outcome data: ICU admission, requirement for inotrope/vasopressor infusion, requirement for non-invasive or invasive ventilation, requirement for renal replacement therapy, requirement for extracorporeal membrane oxygenation (ECMO), final microbiological diagnosis, and mortality.

### Ultrasound techniques

*Cardiac index* will be measured by trans-thoracic echocardiography using a Zonare Z.one (Zonare Medical Systems, Mountain View, CA, USA) and a 3–10 MHz phased array (cardiac) transducer. An apical 5 chamber view of the heart will be obtained and the left-ventricular outflow tract visualised. A 3 mm pulsed-wave doppler sample volume will be gated at the level of the aortic valve and the resulting waveform recorded. The mean velocity-time integral (VTI) over three respiratory cycles will be recorded as the final VTI measurement. Left-ventricular outflow tract (LVOT) diameter will be measured from a parasternal long-axis (PLAX) view in systole. Stroke volume will be calculated as (πr^2^) x velocity-time integral (VTI), and CO as stroke volume SV x HR. CI will be calculated as CO/BSA. Attempts to minimise the impact of inter and intra-observer variability on the study results include: blinding of the ultrasound assessor to the patient identity and timing of the study relative to the timing of fluid bolus administration; and assessment of intra and inter-rater reliability between the PI and the PI and blinded assessor, respectively, for all ultrasound-based assessments.

*Extravascular lung water* will be measured on lung ultrasound using a Zonare Z.one and an 5–14 MHz linear array probe set at a depth of 5 cm. Eight views will be obtained and recorded as described in the Fluid Administration Limited by Lung Sonography (FALLS) protocol [40]. Lung ultrasound cine-loops will be scored in a blinded fashion for the presence of B-lines, confluent B-lines, or complete loss of lung aeration, and used to calculate a lung ultrasound score [41, 42].

*Inferior vena cava collapsibility* will be measured using a Zonare Z.one and a 1–4 MHz curved array transducer. A sub-costal long-axis view of the IVC will be obtained including the IVC-right atrial (RA) junction and confluence of the hepatic veins [43]. A cine-loop recording will be taken over 3 respiratory cycles. IVC collapsibility will be measured at the level of the hepatic veins at end-inspiration and end expiration [44]. IVC collapsibility will be calculated as: maximum - minimum diameter/minimum diameter x 100.

### Sample size, power, and statistical methods

For a precision based sample size estimation based on the continuous outcome variable of change in cardiac output following standardised fluid bolus administration, the following assumptions have been made: a mean increase in cardiac output of 20 % with a standard deviation of 10 %, a precision of the study tool (echocardiogram) of +/− 2 %. For a precision estimate of 5 %, a sample size of 50 would be required. This would also allow for regression analysis of several suspected confounding variables, including age, underlying illness aetiology (respiratory vs non-respiratory), and total volume of fluid previously administered.

### Limitations

Ultrasound-based assessments are user-dependent. As such, they are prone to variability both within and between sonographers. Ultrasound-based assessment of EVLW and fluid responsiveness based on IVC collapsibility have been validated in adult patients but not in children. Age-based physiological differences in children may limit their utility in the study population. Some vital signs recorded (such as capillary refill, conscious state, and respiratory effort) are qualitative and prone to bias. Efforts to minimise the impact of potential bias on the study results include video recording of the patient at each time point and subsequent assessment blinded to the timing of the video recording relative to the timing of fluid bolus administration. At the time of assessment, enrolled patients are undifferentiated and are being treated empirically for sepsis. They may have an alternate final diagnosis. At the time of enrolment, patients are receiving multiple interventions, some of which are distressing and make ultrasound assessment challenging. Interventions other than fluid bolus administration (such as the use of non-invasive or invasive ventilation, or administration of inotropic or vaso-active therapies) may confound the study results. To minimise the effect of other therapies on study results, the study will focus only on the first hour of resuscitation. Any other interventions performed during the study period will be recorded. The study will only recruit patients when the PI is available in the ED. This limits the total number of patients recruited and the time of day when patients are recruited.

## Discussion

Four major questions remain unanswered regarding fluid resuscitation for paediatric sepsis: what is the ideal fluid content, what is the ideal fluid volume, over what time period it should be administered, and what therapeutic targets should be used to titrate therapy. This study will help answer the questions of fluid volume and therapeutic targets. Current paediatric fluid resuscitation guidelines and planned trials are based on the concept of empiric fluid loading using vital signs as a marker of response to treatment. This is despite evidence that the host response to sepsis in children is highly variable and dynamic during the disease course [45–47], and that vital signs are a poor predictor of disease severity or response to treatment [48–51]. As such, using a “one size fits all” approach to fluid resuscitation seems counter-intuitive. Alternatives include fluid responsiveness-based FRT. Data from randomised pilot studies in adults have shown fluid-responsiveness based resuscitation for sepsis allows more targeted fluid resuscitation (more aggressive in fluid responders, less aggressive in fluid non-responders), with an overall reduction in total fluid volume administered compared to standard treatment [52]. Additionally, the results of this study may suggest that routine assessment of EVLW allows for both the benefits and risks of fluid resuscitation to be included in the therapeutic equation for the individual patient. This approach has been described but not evaluated in randomised trials in adults [53].

This study may inform the design of future interventional studies in paediatric sepsis. The use of fluid-responsiveness based resuscitation limited by EVLW observed on lung ultrasound may allow targeted and individualised treatment [54]. This may help avoid the harms from overzealous fluid resuscitation in some children. Such a strategy would need to have a significant impact on patient-centred outcomes for widespread use, due to its implications for resources, training, and infrastructure required in ED’s caring for children.

## Abbreviations

BP, blood pressure; CI, cardiac index; CO, cardiac output; CVP, central venous pressure; ECMO, extracorporeal membrane oxygenation; ED, emergency department; EGDT, early goal-directed therapy; EVLW, extra-vascular lung water; FEAST, fluid expansion as supportive therapy; FRT, fluid resuscitation therapy; GCS, Glasgow Coma Scale; HR, heart rate; IVC, inferior vena cava; PI, principal investigator; RCH, The Royal Children’s Hospital; RR, respiratory rate; ScvO2, central venous oxygen saturation; SIRS, systemic inflammatory response syndrome; SMD, septic myocardial dysfunction; SV, stroke volume
